# 4-[5-Amino-4-(4-fluoro­phen­yl)-3-(pyridin-4-yl)-1*H*-pyrazol-1-yl]benzo­nitrile

**DOI:** 10.1107/S160053681200877X

**Published:** 2012-03-03

**Authors:** Bassam Abu Thaher, Pierre Koch, Dieter Schollmeyer, Stefan Laufer

**Affiliations:** a Faculty of Science, Chemistry Department, Islamic University of Gaza, Gaza Strip, Palestinian Territories; b Institute of Pharmacy, Department of Pharmaceutical and Medicinal Chemistry, Eberhard Karls University Tübingen, Auf der Morgenstelle 8, 72076 Tübingen, Germany; c Department of Organic Chemistry, Johannes Gutenberg-University Mainz, Duesbergweg 10-14, D-55099 Mainz, Germany

## Abstract

In the crystal structure of the title compound, C_21_H_14_FN_5_, the pyrazole ring forms dihedral angles of 38.0 (1), 40.0 (1) and 28.5 (1)° with the directly attached 4-fluoro­phenyl, pyridine and benzonitrile rings, respectively. The crystal packing is characterized by N—H⋯N hydrogen bonds, which result in a two-dimensional network parallel to the *ac*-plane.

## Related literature

For p38α MAP kinase inhibitors having a vicinal 4-fluoro­phen­yl/pyridin-4-yl system connected to a five-membered heterocyclic core, see: Abu Thaher *et al.* (2009 ▶); Peifer *et al.* (2006 ▶). For the inhibitory activity and preparation of the title compound, see: Abu Thaher *et al.* (2012*a* ▶). For related structures, see: Abu Thaher *et al.* (2012*b* ▶,*c* ▶).
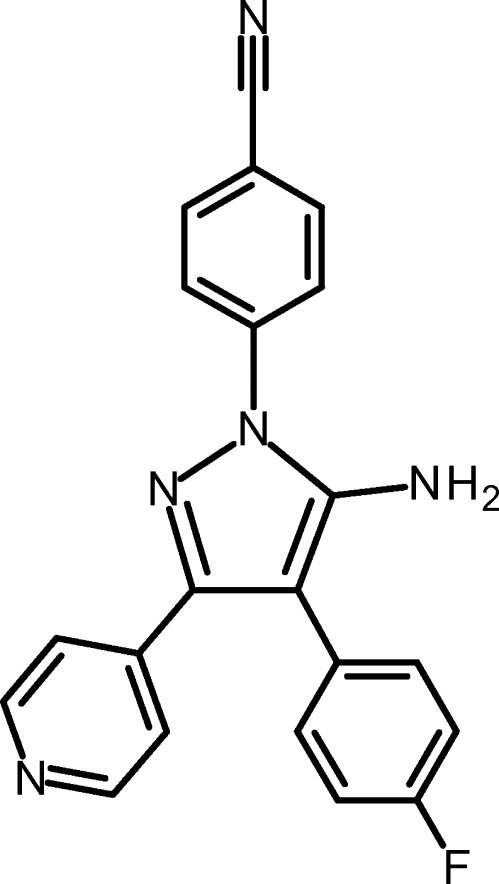


## Experimental

### 

#### Crystal data

C_21_H_14_FN_5_
*M* *_r_* = 355.37
Orthorhombic, 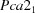

*a* = 10.5189 (5) Å
*b* = 8.1339 (3) Å
*c* = 20.0009 (13) Å
*V* = 1711.27 (15) Å^3^
*Z* = 4
Cu *K*α radiation
μ = 0.76 mm^−1^
*T* = 193 K
0.50 × 0.30 × 0.30 mm

#### Data collection

Enraf–Nonius CAD-4 diffractometer
3163 measured reflections
3059 independent reflections
3005 reflections with *I* > 2σ(*I*)
*R*_int_ = 0.026
3 standard reflections every 60 min intensity decay: 3%

#### Refinement

*R*[*F* ^2^ > 2σ(*F* ^2^)] = 0.038
*wR*(*F* ^2^) = 0.103
*S* = 1.04
3059 reflections
244 parameters
1 restraint
H-atom parameters constrained
Δρ_max_ = 0.17 e Å^−3^
Δρ_min_ = −0.23 e Å^−3^
Absolute structure: Flack (1983 ▶), 1381 Friedel pairs
Flack parameter: −0.17 (18)

### 

Data collection: *CAD-4 Software* (Enraf–Nonius, 1989 ▶); cell refinement: *CAD-4 Software*; data reduction: *CORINC* (Dräger & Gattow, 1971 ▶); program(s) used to solve structure: *SIR97* (Altomare *et al.*, 1999 ▶); program(s) used to refine structure: *SHELXL97* (Sheldrick, 2008 ▶); molecular graphics: *PLATON* (Spek, 2009 ▶); software used to prepare material for publication: *PLATON*.

## Supplementary Material

Crystal structure: contains datablock(s) I, global. DOI: 10.1107/S160053681200877X/im2359sup1.cif

Structure factors: contains datablock(s) I. DOI: 10.1107/S160053681200877X/im2359Isup2.hkl

Supplementary material file. DOI: 10.1107/S160053681200877X/im2359Isup3.cml

Additional supplementary materials:  crystallographic information; 3D view; checkCIF report

## Figures and Tables

**Table 1:** Hydrogen-bond geometry (Å, °)

*D*—H⋯*A*	*D*—H	H⋯*A*	*D*⋯*A*	*D*—H⋯*A*
N6—H6*A*⋯N26^i^	0.88	2.14	2.938 (2)	150
N6—H6*B*⋯N14^ii^	0.86	2.58	3.292 (3)	141

Symmetry codes: (i) 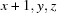; (ii) 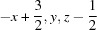.

**Fig. 1.**
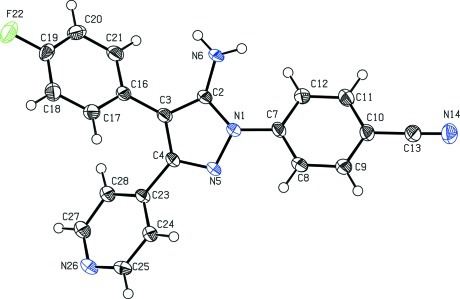
View of the title compound. Displacement ellipsoids are drawn at the 50% probability level. H atoms are depicted as circles of arbitrary size.

**Fig. 2.**
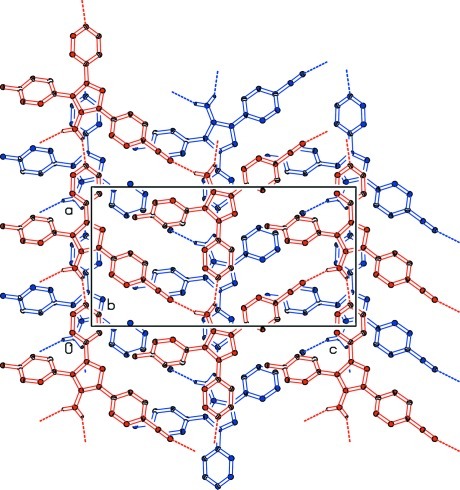
A packing section of the crystal viewed down the b axis. Hydrogen bonds are shown as dashed lines. Separated layers are indicated by different colours.

## supplementary crystallographic information

### Comment

Compounds having a vicinal 4-fluorophenyl/pyridin-4-yl system connected to a five-membered heterocyclic core have been considered to be potential p38α MAP kinase inhibitors (Abu Thaher *et al.* 2009, Peifer *et al.* 2006). We showed that the regioisomeric switch from 3-(4-fluorophenyl)-4-(pyridin-4-yl)-1-(aryl)-1*H*-pyrazol-5-amine to 4-(4-fluorophenyl)-3-(pyridin-4-yl)-1-(aryl)-1*H*-pyrazol-5-amine completely changed the inhibitory profile from p38α MAP kinase to kinases relevant in cancer (Abu Thaher *et al.* 2012*a*). Recently, we reported similar crystal structures (Abu Thaher *et al.* 2012**b*,c*).

In the crystal structure of the title compound (Fig. 1) the pyrazole ring forms dihedral angels of 38.0 (1)°, 40.0 (1)°, 28.5 (1)° with the directly attached 4-fluorophenyl, pyridine and benzonitrile rings, respectively. The 4-fluorophenyl ring encloses dihedral angels of 57.7 (1)° and 22.1 (9)° toward the pyridine and benzonitrile rings, respectively. The pyridine ring is oriented at a dihedral angle of 35.6 (1)° toward the benzonitrile ring.

The crystal packing (Fig. 2) shows that the amino function acts as a hydrogen bond donor of two intermolecular hydrogen bonds formed to two different molecules - one to the nitrogen atom (N26) of the pyridine ring and one to the nitrogen atom (N14) of the nitrile group. The length of the hydrogen bonds is 2.14Å and 2.58 Å, respectively (Table 1). The two hydrogen bonds result in a two dimensional network parallel to the *ac*-plane.

### Experimental

20 mmol of LDA were added to 30 ml of dry THF in a three neck flask and cooled to 194 K. 14 mmol of 4-fluorophenylacetonitril in 10 ml THF were added dropwise and the reaction mixture was stirred for 45 min. 5 mmol of *N*-(4-cyanophenyl)-4-pyridinecarbohydrazonoyl chloride were added slowly and stirring of the reaction mixture was continued for 1 h. After warming to 293 K, 50 ml of water were added to the reaction mixture which then was extracted with ethyl acetate (2x 50 mL). The combined organic layers were dried over Na_2_SO_4_. The organic solution was concentrated to about 5 ml and the product precipitated as pure pale brown crystalls which were washed with diethyl ether. Yield 62%.

### Refinement

Hydrogen atoms attached to carbons were placed at calculated positions with C—H = 0.95 Å (aromatic) or 0.98–0.99 Å (*sp*^3^ C-atom). All H atoms were refined in the riding-model approximation with isotropic displacement parameters (set at 1.2–1.5 times of the *U*_eq_ of the parent atom).

### Crystal data

**Table:**

C_21_H_14_FN_5_	*F*(000) = 736
*M**_r_* = 355.37	*D*_x_ = 1.379 Mg m^−^^3^
Orthorhombic, *P**c**a*2_1_	Cu *K*α radiation, λ = 1.54178 Å
Hall symbol: P 2c -2ac	Cell parameters from 25 reflections
*a* = 10.5189 (5) Å	θ = 65–70°
*b* = 8.1339 (3) Å	µ = 0.76 mm^−^^1^
*c* = 20.0009 (13) Å	*T* = 193 K
*V* = 1711.27 (15) Å^3^	Block, brown
*Z* = 4	0.50 × 0.30 × 0.30 mm

### Data collection

**Table:**

Enraf–Nonius CAD-4 diffractometer	*R*_int_ = 0.026
Radiation source: rotating anode	θ_max_ = 69.9°, θ_min_ = 4.4°
Graphite monochromator	*h* = −12→12
ω/2θ scans	*k* = −9→9
3163 measured reflections	*l* = −24→24
3059 independent reflections	3 standard reflections every 60 min
3005 reflections with *I* > 2σ(*I*)	intensity decay: 3%

### Refinement

**Table:**

Refinement on *F*^2^	Secondary atom site location: difference Fourier map
Least-squares matrix: full	Hydrogen site location: inferred from neighbouring sites
*R*[*F*^2^ > 2σ(*F*^2^)] = 0.038	H-atom parameters constrained
*wR*(*F*^2^) = 0.103	*w* = 1/[σ^2^(*F*_o_^2^) + (0.0752*P*)^2^ + 0.2913*P*] where *P* = (*F*_o_^2^ + 2*F*_c_^2^)/3
*S* = 1.04	(Δ/σ)_max_ < 0.001
3059 reflections	Δρ_max_ = 0.17 e Å^−^^3^
244 parameters	Δρ_min_ = −0.23 e Å^−^^3^
1 restraint	Absolute structure: Flack (1983), 1381 Friedel pairs
Primary atom site location: structure-invariant direct methods	Flack parameter: −0.17 (18)

### Special details

**Table:**

Geometry. All e.s.d.'s (except the e.s.d. in the dihedral angle between two l.s. planes) are estimated using the full covariance matrix. The cell e.s.d.'s are taken into account individually in the estimation of e.s.d.'s in distances, angles and torsion angles; correlations between e.s.d.'s in cell parameters are only used when they are defined by crystal symmetry. An approximate (isotropic) treatment of cell e.s.d.'s is used for estimating e.s.d.'s involving l.s. planes.
Refinement. Refinement of *F*^2^ against ALL reflections. The weighted *R*-factor *wR* and goodness of fit *S* are based on *F*^2^, conventional *R*-factors *R* are based on *F*, with *F* set to zero for negative *F*^2^. The threshold expression of *F*^2^ > σ(*F*^2^) is used only for calculating *R*-factors(gt) *etc*. and is not relevant to the choice of reflections for refinement. *R*-factors based on *F*^2^ are statistically about twice as large as those based on *F*, and *R*- factors based on ALL data will be even larger.

### Fractional atomic coordinates and isotropic or equivalent isotropic displacement parameters (Å^2^)

**Table:**

	*x*	*y*	*z*	*U*_iso_*/*U*_eq_	
N1	0.43666 (14)	0.21998 (19)	0.52903 (8)	0.0245 (3)	
C2	0.46323 (15)	0.2397 (2)	0.46188 (10)	0.0233 (4)	
C3	0.34814 (17)	0.2656 (2)	0.43004 (10)	0.0234 (4)	
C4	0.25715 (16)	0.2607 (2)	0.48226 (9)	0.0236 (4)	
N5	0.30861 (14)	0.23527 (19)	0.54156 (8)	0.0253 (3)	
N6	0.58360 (15)	0.2377 (2)	0.43736 (9)	0.0322 (4)	
H6A	0.6492	0.2547	0.4639	0.048*	
H6B	0.5975	0.2425	0.3949	0.048*	
C7	0.51679 (16)	0.1880 (2)	0.58436 (10)	0.0240 (4)	
C8	0.47853 (17)	0.2416 (3)	0.64701 (11)	0.0307 (4)	
H8	0.4014	0.3013	0.6516	0.037*	
C9	0.55154 (18)	0.2090 (3)	0.70281 (10)	0.0319 (4)	
H9	0.5245	0.2446	0.7457	0.038*	
C10	0.66595 (17)	0.1229 (2)	0.69545 (9)	0.0269 (4)	
C11	0.70398 (19)	0.0686 (2)	0.63280 (10)	0.0309 (4)	
H11	0.7816	0.0102	0.6281	0.037*	
C12	0.62949 (18)	0.0992 (2)	0.57720 (10)	0.0287 (4)	
H12	0.6548	0.0600	0.5345	0.034*	
C13	0.74546 (19)	0.0933 (2)	0.75280 (11)	0.0306 (4)	
N14	0.81025 (18)	0.0714 (3)	0.79805 (10)	0.0402 (4)	
C16	0.33007 (16)	0.3156 (2)	0.35985 (9)	0.0235 (4)	
C17	0.24094 (18)	0.4368 (2)	0.34404 (10)	0.0279 (4)	
H17	0.1915	0.4843	0.3788	0.033*	
C18	0.22306 (19)	0.4892 (2)	0.27863 (11)	0.0315 (4)	
H18	0.1605	0.5693	0.2681	0.038*	
C19	0.29837 (19)	0.4220 (3)	0.22958 (9)	0.0303 (4)	
C20	0.38681 (19)	0.3029 (3)	0.24196 (10)	0.0329 (4)	
H20	0.4369	0.2585	0.2068	0.039*	
C21	0.40156 (19)	0.2480 (3)	0.30763 (10)	0.0294 (4)	
H21	0.4611	0.1634	0.3170	0.035*	
F22	0.28240 (13)	0.47710 (18)	0.16555 (6)	0.0439 (3)	
C23	0.11619 (16)	0.2723 (2)	0.47901 (10)	0.0247 (4)	
C24	0.04902 (17)	0.3515 (3)	0.52898 (10)	0.0301 (4)	
H24	0.0927	0.4043	0.5646	0.036*	
C25	−0.08274 (18)	0.3532 (3)	0.52667 (11)	0.0357 (5)	
H25	−0.1273	0.4093	0.5611	0.043*	
N26	−0.15029 (15)	0.2799 (2)	0.47859 (10)	0.0370 (4)	
C27	−0.08439 (19)	0.2038 (3)	0.43060 (12)	0.0362 (5)	
H27	−0.1306	0.1511	0.3959	0.043*	
C28	0.04695 (18)	0.1972 (3)	0.42836 (11)	0.0301 (4)	
H28	0.0891	0.1422	0.3928	0.036*	

### Atomic displacement parameters (Å^2^)

**Table:**

	*U*^11^	*U*^22^	*U*^33^	*U*^12^	*U*^13^	*U*^23^
N1	0.0145 (6)	0.0350 (8)	0.0241 (8)	−0.0011 (6)	0.0004 (6)	0.0023 (7)
C2	0.0160 (8)	0.0292 (9)	0.0246 (10)	−0.0018 (6)	0.0001 (7)	−0.0011 (7)
C3	0.0177 (8)	0.0267 (8)	0.0259 (9)	−0.0007 (6)	0.0000 (7)	−0.0014 (8)
C4	0.0149 (8)	0.0299 (8)	0.0259 (9)	−0.0019 (6)	−0.0023 (7)	0.0007 (7)
N5	0.0128 (7)	0.0366 (8)	0.0265 (9)	0.0008 (5)	−0.0004 (6)	0.0013 (7)
N6	0.0119 (7)	0.0586 (11)	0.0260 (8)	−0.0022 (7)	0.0012 (6)	−0.0019 (8)
C7	0.0164 (8)	0.0288 (9)	0.0268 (9)	−0.0026 (7)	−0.0035 (7)	0.0019 (8)
C8	0.0175 (8)	0.0447 (11)	0.0298 (10)	0.0032 (7)	0.0008 (8)	−0.0026 (9)
C9	0.0228 (9)	0.0469 (11)	0.0261 (10)	−0.0006 (8)	−0.0003 (7)	−0.0042 (9)
C10	0.0224 (8)	0.0318 (9)	0.0265 (10)	−0.0038 (7)	−0.0041 (7)	0.0022 (8)
C11	0.0252 (9)	0.0357 (10)	0.0320 (10)	0.0068 (8)	−0.0031 (8)	−0.0003 (9)
C12	0.0254 (9)	0.0363 (9)	0.0244 (9)	0.0042 (8)	0.0003 (7)	−0.0028 (8)
C13	0.0260 (8)	0.0365 (10)	0.0291 (9)	−0.0040 (8)	−0.0014 (8)	0.0014 (8)
N14	0.0332 (9)	0.0541 (11)	0.0333 (9)	−0.0074 (8)	−0.0090 (8)	0.0054 (9)
C16	0.0172 (7)	0.0292 (9)	0.0241 (9)	−0.0039 (7)	−0.0008 (6)	−0.0019 (7)
C17	0.0248 (9)	0.0328 (9)	0.0261 (10)	−0.0002 (7)	0.0017 (7)	−0.0024 (8)
C18	0.0297 (10)	0.0336 (10)	0.0314 (10)	−0.0008 (8)	−0.0043 (8)	0.0041 (8)
C19	0.0300 (9)	0.0406 (10)	0.0202 (9)	−0.0136 (8)	−0.0034 (7)	0.0013 (8)
C20	0.0277 (9)	0.0456 (11)	0.0254 (10)	−0.0079 (8)	0.0058 (8)	−0.0078 (9)
C21	0.0202 (9)	0.0367 (10)	0.0313 (10)	−0.0014 (7)	0.0004 (8)	−0.0033 (8)
F22	0.0503 (8)	0.0578 (8)	0.0236 (6)	−0.0142 (6)	−0.0051 (5)	0.0065 (6)
C23	0.0156 (8)	0.0316 (9)	0.0268 (9)	0.0008 (6)	0.0006 (7)	0.0040 (7)
C24	0.0205 (8)	0.0449 (11)	0.0250 (9)	0.0002 (7)	−0.0024 (7)	−0.0022 (9)
C25	0.0213 (9)	0.0538 (12)	0.0318 (10)	0.0055 (8)	0.0055 (8)	−0.0036 (9)
N26	0.0160 (7)	0.0569 (11)	0.0382 (10)	−0.0002 (7)	0.0001 (7)	0.0016 (9)
C27	0.0206 (9)	0.0542 (12)	0.0337 (11)	−0.0049 (8)	−0.0040 (8)	−0.0026 (10)
C28	0.0210 (8)	0.0428 (11)	0.0265 (9)	−0.0017 (7)	−0.0020 (8)	−0.0022 (9)

### Geometric parameters (Å, º)

**Table:**

N1—N5	1.376 (2)	C13—N14	1.147 (3)
N1—C2	1.381 (3)	C16—C17	1.397 (3)
N1—C7	1.415 (2)	C16—C21	1.399 (3)
C2—N6	1.358 (2)	C17—C18	1.389 (3)
C2—C3	1.384 (2)	C17—H17	0.9500
C3—C4	1.417 (3)	C18—C19	1.374 (3)
C3—C16	1.474 (3)	C18—H18	0.9500
C4—N5	1.320 (2)	C19—C20	1.366 (3)
C4—C23	1.487 (2)	C19—F22	1.367 (2)
N6—H6A	0.8816	C20—C21	1.396 (3)
N6—H6B	0.8621	C20—H20	0.9500
C7—C8	1.386 (3)	C21—H21	0.9500
C7—C12	1.395 (3)	C23—C24	1.383 (3)
C8—C9	1.380 (3)	C23—C28	1.389 (3)
C8—H8	0.9500	C24—C25	1.387 (3)
C9—C10	1.400 (3)	C24—H24	0.9500
C9—H9	0.9500	C25—N26	1.336 (3)
C10—C11	1.388 (3)	C25—H25	0.9500
C10—C13	1.440 (3)	N26—C27	1.336 (3)
C11—C12	1.383 (3)	C27—C28	1.383 (3)
C11—H11	0.9500	C27—H27	0.9500
C12—H12	0.9500	C28—H28	0.9500
			
N5—N1—C2	111.40 (14)	C17—C16—C21	117.97 (18)
N5—N1—C7	117.21 (15)	C17—C16—C3	119.80 (17)
C2—N1—C7	131.39 (15)	C21—C16—C3	122.22 (17)
N6—C2—N1	122.58 (16)	C18—C17—C16	121.39 (18)
N6—C2—C3	130.64 (18)	C18—C17—H17	119.3
N1—C2—C3	106.74 (15)	C16—C17—H17	119.3
C2—C3—C4	104.33 (17)	C19—C18—C17	118.20 (18)
C2—C3—C16	126.37 (16)	C19—C18—H18	120.9
C4—C3—C16	128.50 (17)	C17—C18—H18	120.9
N5—C4—C3	112.93 (16)	C20—C19—F22	119.08 (19)
N5—C4—C23	117.26 (16)	C20—C19—C18	123.03 (18)
C3—C4—C23	129.75 (18)	F22—C19—C18	117.90 (18)
C4—N5—N1	104.59 (15)	C19—C20—C21	118.24 (19)
C2—N6—H6A	120.7	C19—C20—H20	120.9
C2—N6—H6B	120.9	C21—C20—H20	120.9
H6A—N6—H6B	117.0	C20—C21—C16	121.13 (19)
C8—C7—C12	120.14 (18)	C20—C21—H21	119.4
C8—C7—N1	118.43 (16)	C16—C21—H21	119.4
C12—C7—N1	121.39 (18)	C24—C23—C28	117.64 (16)
C9—C8—C7	120.58 (17)	C24—C23—C4	120.48 (17)
C9—C8—H8	119.7	C28—C23—C4	121.79 (17)
C7—C8—H8	119.7	C23—C24—C25	119.40 (18)
C8—C9—C10	119.30 (19)	C23—C24—H24	120.3
C8—C9—H9	120.4	C25—C24—H24	120.3
C10—C9—H9	120.4	N26—C25—C24	123.45 (19)
C11—C10—C9	120.12 (18)	N26—C25—H25	118.3
C11—C10—C13	119.92 (17)	C24—C25—H25	118.3
C9—C10—C13	119.95 (18)	C27—N26—C25	116.60 (16)
C12—C11—C10	120.38 (18)	N26—C27—C28	124.0 (2)
C12—C11—H11	119.8	N26—C27—H27	118.0
C10—C11—H11	119.8	C28—C27—H27	118.0
C11—C12—C7	119.46 (19)	C27—C28—C23	118.87 (19)
C11—C12—H12	120.3	C27—C28—H28	120.6
C7—C12—H12	120.3	C23—C28—H28	120.6
N14—C13—C10	178.9 (2)		
			
N5—N1—C2—N6	176.84 (17)	C8—C7—C12—C11	−1.5 (3)
C7—N1—C2—N6	−3.2 (3)	N1—C7—C12—C11	−179.04 (17)
N5—N1—C2—C3	−1.1 (2)	C2—C3—C16—C17	−136.16 (19)
C7—N1—C2—C3	178.89 (17)	C4—C3—C16—C17	32.0 (3)
N6—C2—C3—C4	−177.24 (19)	C2—C3—C16—C21	42.5 (3)
N1—C2—C3—C4	0.45 (19)	C4—C3—C16—C21	−149.42 (18)
N6—C2—C3—C16	−6.8 (3)	C21—C16—C17—C18	0.1 (3)
N1—C2—C3—C16	170.87 (17)	C3—C16—C17—C18	178.76 (17)
C2—C3—C4—N5	0.3 (2)	C16—C17—C18—C19	−1.9 (3)
C16—C3—C4—N5	−169.81 (17)	C17—C18—C19—C20	2.1 (3)
C2—C3—C4—C23	−176.61 (17)	C17—C18—C19—F22	−178.44 (16)
C16—C3—C4—C23	13.2 (3)	F22—C19—C20—C21	−179.87 (17)
C3—C4—N5—N1	−0.97 (19)	C18—C19—C20—C21	−0.4 (3)
C23—C4—N5—N1	176.39 (15)	C19—C20—C21—C16	−1.5 (3)
C2—N1—N5—C4	1.26 (19)	C17—C16—C21—C20	1.7 (3)
C7—N1—N5—C4	−178.71 (15)	C3—C16—C21—C20	−176.99 (18)
N5—N1—C7—C8	−27.3 (2)	N5—C4—C23—C24	38.6 (3)
C2—N1—C7—C8	152.71 (19)	C3—C4—C23—C24	−144.6 (2)
N5—N1—C7—C12	150.24 (17)	N5—C4—C23—C28	−137.84 (19)
C2—N1—C7—C12	−29.7 (3)	C3—C4—C23—C28	39.0 (3)
C12—C7—C8—C9	0.5 (3)	C28—C23—C24—C25	0.0 (3)
N1—C7—C8—C9	178.05 (18)	C4—C23—C24—C25	−176.60 (18)
C7—C8—C9—C10	0.9 (3)	C23—C24—C25—N26	0.8 (3)
C8—C9—C10—C11	−1.1 (3)	C24—C25—N26—C27	−0.8 (3)
C8—C9—C10—C13	177.70 (19)	C25—N26—C27—C28	0.1 (3)
C9—C10—C11—C12	0.1 (3)	N26—C27—C28—C23	0.6 (4)
C13—C10—C11—C12	−178.77 (19)	C24—C23—C28—C27	−0.6 (3)
C10—C11—C12—C7	1.3 (3)	C4—C23—C28—C27	175.92 (19)

### Hydrogen-bond geometry (Å, º)

**Table:**

*D*—H···*A*	*D*—H	H···*A*	*D*···*A*	*D*—H···*A*
N6—H6*A*···N26^i^	0.88	2.14	2.938 (2)	150
N6—H6*B*···N14^ii^	0.86	2.58	3.292 (3)	141

Symmetry codes: (i) *x*+1, *y*, *z*; (ii) −*x*+3/2, *y*, *z*−1/2.
